# Bleeding Complications and Challenges in Establishing a Diagnosis of AL Amyloidosis of the Lymph Nodes: A Case Report

**DOI:** 10.1155/crh/4105506

**Published:** 2026-11-15

**Authors:** Hasan Aladili, Stacy Epstein, Khuda Dad Khan

**Affiliations:** ^1^ Norton Healthcare Inc. Louisville Kentucky USA

## Abstract

Light‐chain amyloidosis (AL) is an uncommon, multisystem disorder in which lymphadenopathy is a rare presentation in the absence of other organ involvement. Establishing a diagnosis can be extremely challenging due to bleeding complications stemming from coagulopathy and capillary fragility caused by amyloid vascular deposition. Bleeding complications lead to higher morbidity and mortality and lower diagnostic yield. We present a case of a thirty‐six‐year‐old man who required prolonged hospitalization to establish the diagnosis of AL amyloidosis of the lymph nodes due to severe bleeding after surgical interventions. He underwent an endoscopic ultrasound (US) with fine‐needle aspiration (FNA) of mesenteric lymph nodes and an endobronchial US with FNA of paratracheal lymph nodes. Both pathology reports showed mature lymphocytes admixed with blood and fibrin. Large hemoperitoneum and hemorrhagic shock ensued after the mesenteric lymph node biopsy. A subsequent excisional biopsy of an inguinal lymph node was normal. A surgical thoracoscopy with mediastinal node biopsy became necessary and was complicated by hemothorax requiring a thoracostomy tube insertion, a transfer to the intensive care unit (ICU), and an emergent video‐assisted thoracoscopy (VATS) due to uncontrollable bleeding. During the hospital stay, the patient required multiple transfusions of blood products and coagulation agents to achieve hemostasis and hemodynamic stability. The diagnosis of amyloidosis was made after the mediastinal lymph node biopsy revealed Congo red positive tissue deposits with characteristic apple‐green birefringence. After confirmation of the diagnosis, treatment was initiated with daratumumab, bortezomib, and dexamethasone. Subsequently, the patient was released from the hospital. He remains on this regimen in the outpatient setting and has shown a positive therapeutic response. He is now being considered for high‐dose chemotherapy followed by autologous stem cell transplant.

## 1. Background

Systemic immunoglobulin light‐chain (AL) amyloidosis is a multisystem disorder characterized by the extracellular deposition of misfolded monoclonal immunoglobulin light chains as amyloid fibrils, resulting in progressive organ dysfunction. Although nearly any organ may be affected, the heart and kidneys are most commonly involved, with hepatic, neurologic gastrointestinal, and soft‐tissue involvement also occurring [1, 2]. The amyloidogenic light chains are produced by an underlying clonal plasma‐cell or, less commonly, B‐cell population and may be either kappa or lambda restricted [1, 3]. Because the clinical manifestations of AL amyloidosis are heterogenous and often nonspecific, diagnostic delays are common and may result in presentation with advanced organ dysfunction [1, 2]. Diagnosis requires the demonstration of amyloid deposits in tissue, classically by Congo red staining with characteristic apple‐green birefringence under polarized light, followed by accurate amyloid typing to establish the precursor protein [1, 4]. Serum‐free light‐chain assays, along with serum protein electrophoresis (SPEP) and immunofixation electrophoresis are imperative to support the tissue‐based diagnosis. Additionally, searching for clonal plasma cells when examining the bone marrow is necessary to reveal the root cause of amyloidosis.

AL amyloidosis, exclusively affecting the lymph nodes, in the absence of other organ involvement, is rarely encountered in clinical practice. Case reports have shown primary amyloidosis involving submandibular lymph nodes [5]. Similar cases have been reported showing amyloidosis affecting celiac and parapancreatic lymph nodes [6] as well. In such cases, lymph node biopsy provides the only potential diagnostic site [7].

## 2. Case Presentation

A thirty‐six‐year‐old man presented to the emergency department with progressive right‐sided abdominal pain present for the previous 3 months and associated with early satiety, decreased oral intake, and weight loss. His medical history was remarkable for right inguinal hernia and a family history of unspecified cancer.

Six months prior to this admission, he was hospitalized at an academic tertiary care facility after spontaneous hemoperitoneum was found on computed tomography (CT) of the abdomen and pelvis. CT imaging also revealed left mesenteric masses; therefore, an endoscopic ultrasound (US) and biopsy were planned but ultimately not completed due to the patient leaving the hospital against medical advice. One week prior to this hospital admission, he visited a general surgeon for evaluation of a right inguinal hernia. During that time, a CT scan redemonstrated the mesenteric masses with minimal free pelvic fluid. On the day of admission, he presented to an urgent care center complaining of abdominal pain and was subsequently referred to the emergency department due to the recent abnormal CT findings.

On the day of admission, he had normal vital signs, appeared well, and in no acute distress. His cardiovascular and pulmonary exams were unremarkable. His abdomen was soft and mildly tender to palpation, without rigidity guarding or hepatosplenomegaly. Additionally, a right‐sided reducible inguinal hernia and a prominent but soft left inguinal lymph node were noted.

His initial blood work revealed a hemoglobin of 9.9 g/dL (13.5–17.5 g/dL) with a mean corpuscular volume (MCV) of 72.9 fL (80–100 fL) and platelets count of 326 10∗3/μL (140–440 10∗3/μL). A comprehensive metabolic panel showed blood creatine of 0.83 mg/dL (0.73–1.18 mg/dL), total protein of 6.8 g/dL (6.4–8.2 g/dL), albumin of 4.6 g/dL (3.5–5.2 g/dL), and calcium of 9.6 mg/dL (8.4–10.2 mg/dL). Also, it showed proteinuria of 30 mg/dL (negative). High sensitivity Troponin‐I was 29 ng/L (0–35 ng/L). INR was 1.3. The following coagulation studies were evaluated throughout the hospital stay as part of the workup for his recurrent bleeding complications: PTT 29.6 s (25.1–36.5 s), Fibrinogen 227 mg/dL (200–400 mg/dL), Factor II Activity 90.7% (79%–131%), Factor V Activity 122.2% (62%–139%), Factor VII Activity 90.2% (50%–129%), Factor VIII Activity 111% (50%–150%), Factor IX Activity 105.1% (50%–150%), Factor X Activity 81.9% (77% to 131%), PFA‐100 Collagen/, Epinephrine 189 s (82 to 150 s), Collagen/, ADP 158 s (62–100 s), Von Willebrand Factor (vWF) Activity 167% (40%–163%), C3 Complement 99 mg/dL (88–203 mg/dL), and C4 Complement 31 mg/dL (13–52 mg/dL).

CT of the abdomen and pelvis with intravenous contrast demonstrated small free pelvic fluid with two left mesenteric masses measuring 24 × 23 mm (Figure 1) and 27 × 18 mm (Figure 2). CT of the chest with intravenous contrast showed right paratracheal lymphadenopathy measuring 30 × 39 mm (Figure 3) and a prominent subcarinal lymph node measuring 20 mm.

**FIGURE 1:**
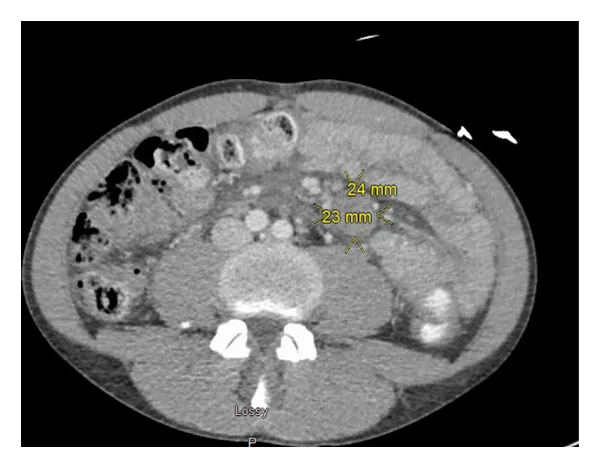
CT of the abdomen and pelvis showing left mesenteric mass 24 × 23 mm.

**FIGURE 2:**
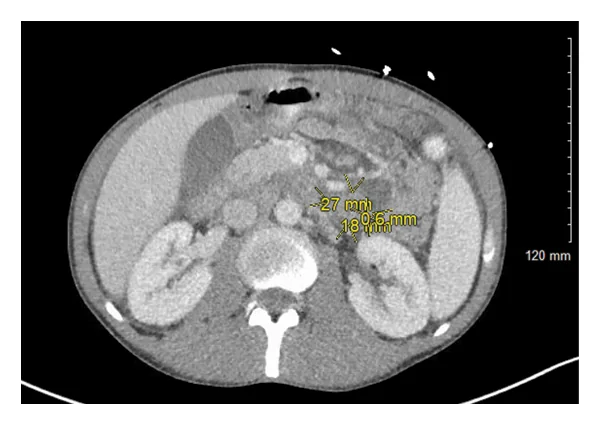
CT of the abdomen and pelvis showing left mesenteric mass 27 × 18 mm.

**FIGURE 3:**
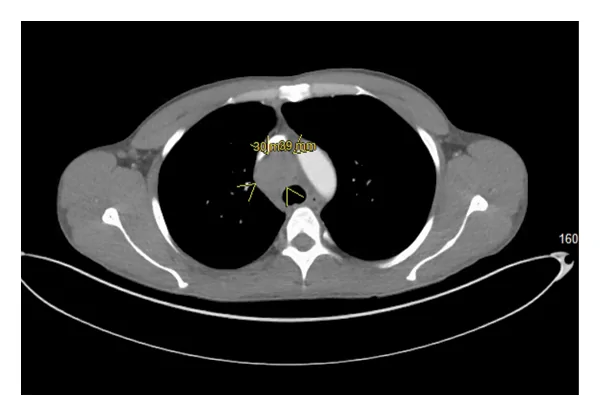
CT of the chest showing right paratracheal lymphadenopathy 30 × 39 mm.

To establish a tissue diagnosis, an endoscopic US with fine‐needle aspiration (FNA) of the mesenteric mass was performed. Pathology showed mature lymphocytes admixed with blood and fibrin. The following day, the patient developed hemorrhagic shock with evidence of large hemoperitoneum on CT imaging of the abdomen and pelvis (Figure 4). He was transfused with multiple units of packed red blood cells (pRBC). An abdominal aortogram was performed by interventional radiology, but no active extravasation was identified.

**FIGURE 4:**
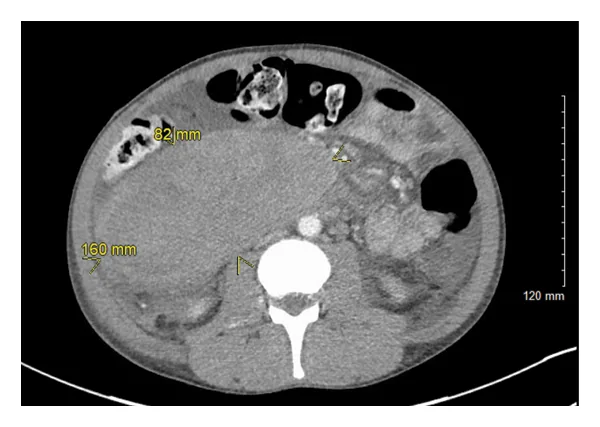
CT abdomen and pelvis showing postbiopsy hemoperitoneum.

Once the patient was stabilized, an endobronchial US with FNA of the paratracheal lymph nodes was performed. Pathology revealed mature lymphocytes mixed with blood and fibrin, without evidence of carcinoma or granuloma. Flow cytometry was within normal limits. Subsequently, a left inguinal lymph node excisional biopsy was performed by general surgery. This revealed a benign lymph node with mildly fibrotic capsule and no evidence of malignancy. Flow cytometry was again unremarkable. At this juncture, thoracic surgery was consulted for a surgical mediastinal biopsy, and a right‐sided robotic‐assisted thoracoscopy with mediastinal biopsy ensued. A drainage thoracostomy tube was present briefly post operatively and was soon removed uneventfully. The following day, the patient complained of right sided rib and back pains. He was noted to be bleeding from the site of the thoracostomy tube that had been removed. Hemoglobin dropped by 1.1 g/dL. An x‐ray of the chest revealed a moderate pleural effusion (Figure 5), and a subsequent CT of the chest showed a right hemothorax with suspected bleeding mediastinal lymph node (Figure 6). The patient developed hypotension and tachycardia requiring transfer to the intensive care unit, multiple units of pRBC transfusion, and a reinsertion of thoracostomy tube at bedside. Seventeen‐hundred milliliters of dark blood in the chamber of the thoracostomy tube were collected over the following 12 h. The patient received a transfusion of one unit of platelets due to abnormal platelet function, one unit of fresh frozen plasma (FFP), an infusion of cryoprecipitate, and a dose of tranexamic acid (TXA). The patient was taken to the operating room emergently for a right video‐assisted thoracoscopy with evacuation of fibrinous hemothorax. He received a dose of DDAVP and an additional dose of TXA postoperatively, and the output from postoperative thoracostomy tube diminished and the tube was successfully removed.

**FIGURE 5:**
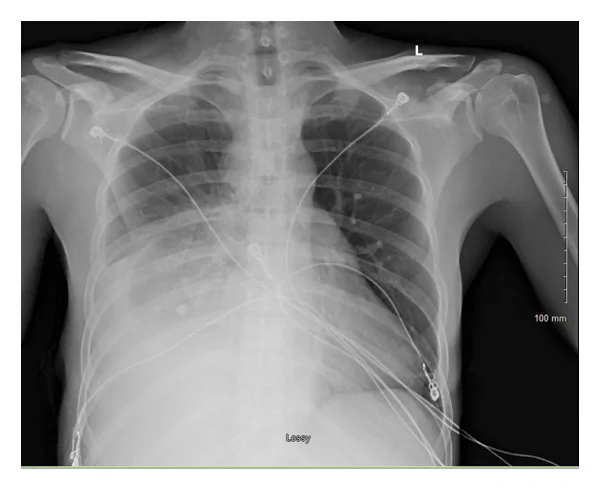
Chest x‐ray showing right hemothorax and bulging right paratracheal hematoma/adenopathy.

**FIGURE 6:**
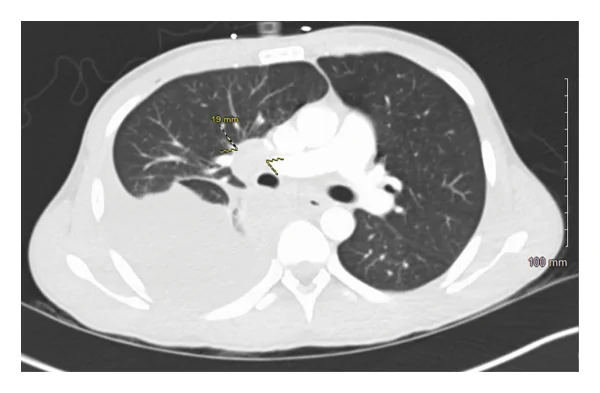
CT of the chest showing right hemothorax with bleeding mediastinal lymph node.

Lymph node biopsy was positive for Congo red stain (Figures 7 and 8). Liquid chromatography and tandem mass spectrometry performed at Mayo clinic laboratories detected a peptide profile consistent with AL‐(lambda)‐type amyloid deposition. A bone marrow biopsy was performed and showed involvement by plasma cell neoplasm (9% plasma cells by aspirate differential, 15%–20% plasma cells by immunohistochemistry, monoclonal lambda by flow cytometry) and focal perivascular amyloid deposits identified by Congo red stain. Clonal plasma cells showed positivity for CD20 and BCL1 by flow cytometry and immunohistochemistry, respectively, which often correlates with *t* (11; 14). A FISH panel for myeloma, however, failed to show that translocation and instead was positive for deletion of TP 53 and monosomy 17.

**FIGURE 7:**
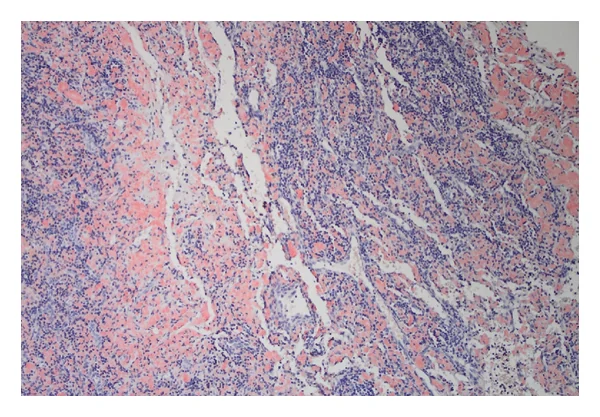
Amyloid deposits are highlighted as deep red‐salmon color in lymph node tissue by light microscopy of Congo red stain (original magnification × 10).

**FIGURE 8:**
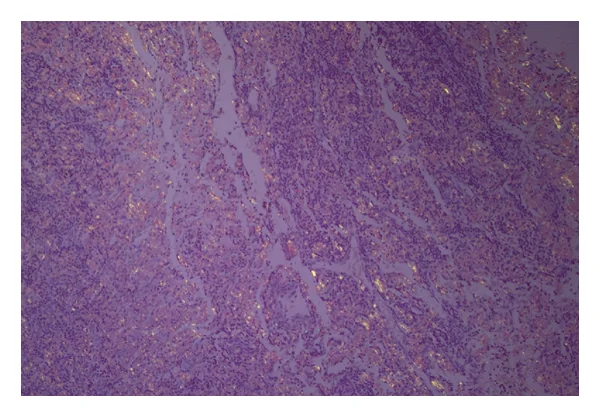
Amyloid deposits on Congo red stain demonstrating characteristic apple green birefringence under polarized light (original magnification × 10).

SPEP and immunofixation revealed the following: Free kappa 12.82 mg/L (3.30–19.40 mg/L), Free Lambda 136.62 mg/L (5.71–26.30 mg/L), and Free Kappa/Lambda Ratio 0.09 mg/L (0.26–1.65 mg/L).

A transthoracic echocardiogram was performed and showed severe left ventricular hypertrophy with a normal ejection fraction 55%–60%, without suspicious findings of amyloid involvement. Further workup for infiltrative disease was contemplated; however, a cardiac MRI could not be performed in the acute hospital setting while the patient was in the intensive care unit (ICU). An endomyocardial biopsy was considered to have prohibitive risk of bleeding complications in this patient who had severe bleeding diathesis and was already requiring multiple interventions for hemostasis. Lack of proteinuria and normal kidney size on CT of the abdomen and pelvis made renal involvement less likely, and a kidney biopsy was not performed due a prohibitive risk of bleeding. Multiple CT scans filed to demonstrate skeletal findings of multiple myeloma. Positron emission tomography (PET) scan and whole body magnetic resonance imaging (MRI) were planned upon outpatient follow up as PET was not available in the inpatient setting and a whole body MRI was not clinically appropriate in the intensive care setting.

The following laboratory tests were evaluated and found to be within the normal range throughout the course of hospital stay: CEA, CA 19–9, AFP, beta‐HCG, beta‐2‐micrglobulin, HIV, blood cultures, AFB, Microgen CMV and EBV PCR, Fungitell, fungal serologies, IgG4, RF, MPO IgG, Proteinase 3 IgG, HHV‐8, Chromogranin A, Hepatitis panel, ANA cascade, and ACE level.

After confirmation of the diagnosis of AL amyloidosis in association with plasma cell dyscrasia, treatment options were discussed with the patient, including high‐dose chemotherapy and stem cell transplant for definitive treatment. The standard induction therapy of daratumumab, bortezomib, cyclophosphamide, and dexamethasone (Dara‐VCd) was recommended to the patient; however, he was reluctant to take chemotherapy due to potential toxicity of nausea and vomiting and , therefore, cyclophosphamide was withheld. The patient was promptly treated with daratumumab, bortezomib, and dexamethasone. He continues to receive this treatment in the outpatient infusion center. He reported episodes of epistaxis during office visits; however, his hemoglobin has remained stable at 11.6 g/dL (13.5–17.5 g/dL).

He has demonstrated a favorable therapeutic response to this regimen and is currently undergoing evaluation for high‐dose chemotherapy followed by stem cell transplant.

## 3. Discussion

AL amyloidosis is a rare, multisystem disorder characterized by the presence of amyloid deposition in the target organs causing progressive organ failure. Amyloid light chains are produced by low‐grade expansion of clonal plasma cells as demonstrated by bone marrow biopsy in our patient. Although our patient could have other organ involvement, his critical condition while hospitalized and major bleeding complications precluded further organ system evaluation. Plasmacytosis and other clinical findings were not sufficient to meet a diagnosis of multiple myeloma. Furthermore, management of this patient would not significantly differ regardless of the exact classification.

Major bleeding complications increase morbidity and mortality in AL amyloidosis. In one case series, major bleeding was reported in 9% of the patients and mortality occurred in 19% of those affected by hemorrhagic complications [8]. Despite this, the coagulation dysregulation and vascular fragility resulting in these complications have not been well studied and the pathogenesis of bleeding dyscrasia remains poorly understood. Multiple potential underlying mechanisms have been proposed. In a subset of patients, clotting abnormalities such as acquired Factor X deficiency, increased intravascular coagulation, and increased fibrinolysis have all been reported [8]. There was no evidence of coagulopathy in our case. The elevated serum vWF levels ruled out von Willebrand disease and likely reflected endothelial injury and inflammation noted in patients with amyloidosis leading to vascular fragility [8].

The patient required transfusion with nine units of pRBC, a unit of platelets, a unit of FFP, an infusion of DDAVP, and two doses of TXA in addition to therapy directed at plasma cells to achieve hemostasis. This highlights the need for improved diagnostic approaches and further guidance by the hematologic society on specific protocols of therapeutic agents to mitigate bleeding. The rarity of this condition and heterogenous presentation, however, makes this difficult to establish.

Administration of DDAVP and TXA in the perioperative period while pursuing tissue diagnosis can perhaps form the building blocks upon which hemostasis can be achieved for similar cases in the future. Transfusion of adequate blood products was necessary to maintain hemodynamic stability, which could pose an additional layer of complications for patients with heart failure due to cardiac amyloid involvement who are intolerant of large volume infusions. Ultimately prompt and definitive therapy directed at plasma cells played a major role in the successful discharge of this patient from the hospital.

## Author Contributions

Hasan Aladili contributed to data collection, manuscript drafting, and revision. Stacy Epstein contributed to clinical management and critical revision of the manuscript. Khuda Dad Khan contributed to clinical management, critical revision of the manuscript, and supervision.

## Funding

The authors received no financial support for the research, authorship, and/or publication of this article.

## Disclosure

All authors have read and approved the final version of the manuscript. Hasan Aladili takes complete responsibility for the integrity and accuracy of data presented.

## Ethics Statement

No IRB approval was required for this single case report; however, informed consent from the patient was obtained according to institutional guidelines.

## Conflicts of Interest

The authors declare no conflicts of interest.

## Data Availability

Data sharing is not applicable to this article as no datasets were generated or analyzed during the current study.
